# The von Hippel-Lindau Tumor Suppressor Protein Promotes c-Cbl-Independent Poly-Ubiquitylation and Degradation of the Activated EGFR

**DOI:** 10.1371/journal.pone.0023936

**Published:** 2011-09-16

**Authors:** Liang Zhou, Haifeng Yang

**Affiliations:** Department of Cancer Biology, Lerner Research Institute, Cleveland Clinic, Cleveland, Ohio, United States of America; King's College London, United Kingdom

## Abstract

Somatic mutations or reduced expression of the von Hippel-Lindau (VHL) tumor suppressor occurs in the majority of the clear cell renal cell carcinoma (ccRCC) and is a causal factor for the pathogenesis of ccRCC. pVHL was reported to suppress the oncogenic activity of Epidermal Growth Factor Receptor (EGFR) by reducing the expression of the EGFR agonist TGF-α and by reducing the translation efficiency of EGFR itself. Furthermore, it was reported that pVHL down-regulates activated EGFR by promoting efficient lysosomal degradation of the receptor. These modes of negative regulation of EGFR by pVHL were dependent on Hypoxia Inducible Factor (HIF). In this study, we report that HIF was not the only factor stabilizing the activated EGFR in *VHL*-deficient ccRCC cells. Down-regulation of endogenous HIF in these cells had little effect on the turnover rates of the activated EGFR. Furthermore, neither pretreatment with lysomomal inhibitors pretreatment nor down-regulation of c-Cbl, a major E3 ubiquitin ligase that targets the activated EGFR for lysosomal degradation, significantly increased the stabilities of EGFR in VHL-expressing ccRCC cells. In contrast, pretreatment with proteasomal inhibitors extended EGFR lifetime and led to similar EGFR half-lives in VHL-expressing and VHL-deficient ccRCC cells. Down-regulation of c-Cbl in VHL-deficient ccRCC cells revealed that the c-Cbl and pVHL collaborated to down-regulate the activated EGFR. Finally, we found that pVHL promoted the poly-ubiquitylation of the activated EGFR, and this function was c-Cbl-independent. Thus these results indicate that pVHL limits EGFR signaling by promoting c-Cbl-independent poly-ubiquitylation of the activated receptor, which likely results in its degradation by proteasome.

## Introduction

Approximately 75% of kidney cancers are characterized as clear cell renal cell carcinoma (ccRCC). von Hippel-Lindau (*VHL*) tumor suppressor gene inactivation plays a causal role in the pathogenesis of ccRCC. Approximately 70% of sporadic ccRCC tumors have biallelic inactivation of *VHL* due to either mutation, deletion, or hypermethylation of the promoter that reduces *VHL* expression [1], [2]. Inherited germline mutations in *VHL* predispose hereditary kidney cancer patients, to earlier onset bilateral kidney cancer than sporadic kidney cancer patients, since the loss of the remaining wild type allele occurs more readily than the loss of two alleles. pVHL, the protein product of the *VHL* tumor suppressor gene, is responsible for substrate recognition by an E3 ubiquitin ligase complex that consists of pVHL, CUL2, Elongin B and C, and Rbx1 [3]. This complex adds poly-ubiquitin chains onto the α subunits of the heterodimeric transcription factor hypoxia-inducible factor (HIF), leading to proteasome-mediated degradation of HIF [4]. pVHL is also involved in HIF-independent biological processes including NF-κB activity inhibition [5], maintenance of chromosome stability [6], cilia production [7], and stabilization of RNA polymerase II subunit 1 [8].

The HIF transcription factor is composed of two subunits: the oxygen-sensitive α subunits (HIF1α, HIF2α, and HIF3α) and the constitutively expressed HIF1β subunit (also called the arylhydrocarbon nuclear translocator [ARNT]) [9]. pVHL only binds to HIFα if one of two prolyl residues is hydroxylated by members of the EglN family (also called PHDs or HPHs) [10], [11], [12], [13]. These enzymes need molecular oxygen, Fe(II) and 2-oxo-glutarate for proper function. Under normal oxygen tension (normoxia), the α subunits of HIF are prolyl-hydoxylated, poly-ubiquitylated and destroyed by proteasome. During oxygen deprivation (hypoxia), the α subunits are not prolyl-hydroxylated, escape recognition by pVHL and evade degradation, and heterodimerize with HIF1β. The HIF complex enters the nucleus and regulates the expression of hundreds of target genes by binding to hypoxia-response elements (HRE) [14]. Activation of HIF leads to dramatic changes in cellular physiology: a metabolic shift to anerobic glycolysis, increased secretion of pro-angiogenesis factors, remodeling of the extracellular matrix, and resistance to apoptosis and increased mobility. These changes help the cells to cope with the short-term and long-term consequences of oxygen deprivation, a form of environmental stress. HIF is constitutively activated in the *VHL*-defective ccRCC tumors despite the fact that they are well-oxygenated, since enhanced angiogenesis as a result of increased secretion of pro-angiogenesis factors is a prominent feature of ccRCC. In pre-clinical models of ccRCC, HIF suppression is necessary and sufficient for VHL-dependent suppression of tumor growth [15] [16], [17]. This suggests that inhibiting the activities of HIF or its critical target genes is clinically important for treatment of ccRCC. In keeping with the pre-clinical models, drugs that inhibit the kinase activity of the receptors for Vascular Endothelial Growth Factor (VEGF), a critical HIF target gene, have been proved to be clinically effective and have become major therapeutic agents for treating kidney cancer [18].

It is known that HIF can activate EGFR to promote tumor growth [19], [20]. In *VHL*-defective ccRCC cells, HIF2α induces expression of transforming growth factor-α (TGF-αan agonist to EGFR, and stimulates cell proliferation through an autocrine loop [19]. At the same time, constitutively active HIF2α also enhances the efficiency of EGFR mRNA translation [20]. Increased EGFR expression and elevated TGF-α collectively promote autonomous growth (cellular growth in the absence of stimulating growth factors), a hallmark of cancer. Stable suppression of EGFR by shRNA prevents serum-free growth of *VHL*-defective ccRCC cells *in vitro*, and retards the tumor growth of these cells for extended periods in xenograft models, without affecting HIF2αfunctions [21], [22]. This indicates that the EGFR is critical for the tumorigenesis of *VHL*-defective ccRCC cells and is a credible therapeutic target in kidney cancer.

EGFR is implicated in the development of many human cancers, as activating mutations of EGFR have been identified in human glioblastoma, non-small cell lung carcinomas (NSCLC), and colon cancer. Also, inhibitors of EGFR kinase activity elicit cell death and tumor shrinkage in NSCLC patients with certain EGFR mutations [23]. EGFR consists of an extracellular region with two ligand-binding domains, an extracellular juxta-membrane region, a hydrophobic trans-membrane domain, a cytoplasmic tyrosine kinase domain and c-terminal tyrosine residues that are sites of receptor phosphorylation. Upon ligand binding, receptors homo- or hetero-dimerize, trans-phosphorylate the c-terminal tyrosine residues, recruit signaling molecules to these phosphorylated residues and activate downstream effectors and biological responses [24]. As Ras/Raf/MEK/ERK and PI3K/PDK1/Akt1 are two major downstream effector pathways of activated EGFR that promote both cellular proliferation and resistance to apoptosis [25], failure to turn off the activated EGFR can drive oncogenesis.

Endocytosis and lysosome-mediated degradation are major mechanisms to down-regulate the activated EGFR. The ubiquitin ligase c-Cbl ubiquitylates phosphorylated EGFR [26]. c-Cbl binds to EGFR either directly to phosphorylated Y1045 [27] or through its association with another EGFR-interacting protein Grb2 [28]. Ubiquitylation is sufficient [29] but not necessary [30] for its endocytosis, because multiple redundant mechanisms participate in the endocytosis of activated EGFR [31]. Interestingly, although the ubiquitylation of EGFR is dispensable for endocytosis, it is absolutely required for the efficient turnover of the EGFR protein after its activation [30]. Endocytosed EGFR moves from early endosome to the multi-vesicular body before it is finally sorted to lysosome for degradation [32]. Controversy exists on the modes of the EGFR ubiquitylation, as EGFR has been reported to become both mono-ubiquitylated and poly-ubiquitylated. c-Cbl promotes mono-ubiquitylation on multiple lysine residues of EGFR, which is sufficient for EGFR endocytosis and degradation [29], [33], However, mass-spectrometric and western blot analyses have suggested that a fraction of activated EGFR is poly-ubiquitylated [34], [35]. Currently, however, no specific E3 that promotes poly-ubiquitylation of the activated EGFR has been identified.

Recently it was reported that pVHL was essential for the clearance of activated EGFR [36]. The proposed mechanism was that constitutively active HIF suppressed the expression of Rabaptin-5 at the transcriptional level. As Rabaptin-5 was critical for Rab5-mediated endosome fusion, reduced expression of Rabaptin-5 led to delayed EGFR sorting to the late endosome and lysosome, hence retarding degradation. This explanation predicted that delayed turnover of activated EGFR in *VHL*-defective ccRCC cells was due to high endogenous levels of HIF α subunits. In this study, however, we report that endogenous HIF was not the only cause of delayed EGFR turnover in *VHL*-defective ccRCC cells. Furthermore, we found that down-regulation of the activated EGFR in these cells was likely mediated by both proteasomal and lysosomal degradation. In addition, loss of both c-Cbl and VHL had an additive effect on EGFR stability, suggesting that these ubiquitin ligases collaborated to down-regulate activated EGFR. Finally, we found that pVHL promoted the poly-ubiquitylation of the activated EGFR, and this persisted in the absence of c-Cbl. Thus in ccRCC cells, pVHL promotes the poly-ubiquitylation of the activated EGFR that is independent of c-Cbl, and this leads to proteasomal degradation of activated EGFR. In *VHL*-defective ccRCC cells, the prolonged signaling of the activated EGFR likely contributes to tumor growth.

## Results

### Activated EGFR had higher stability in VHL-deficient cells than in VHL-expressing ccRCC cells

**Figure 1:**
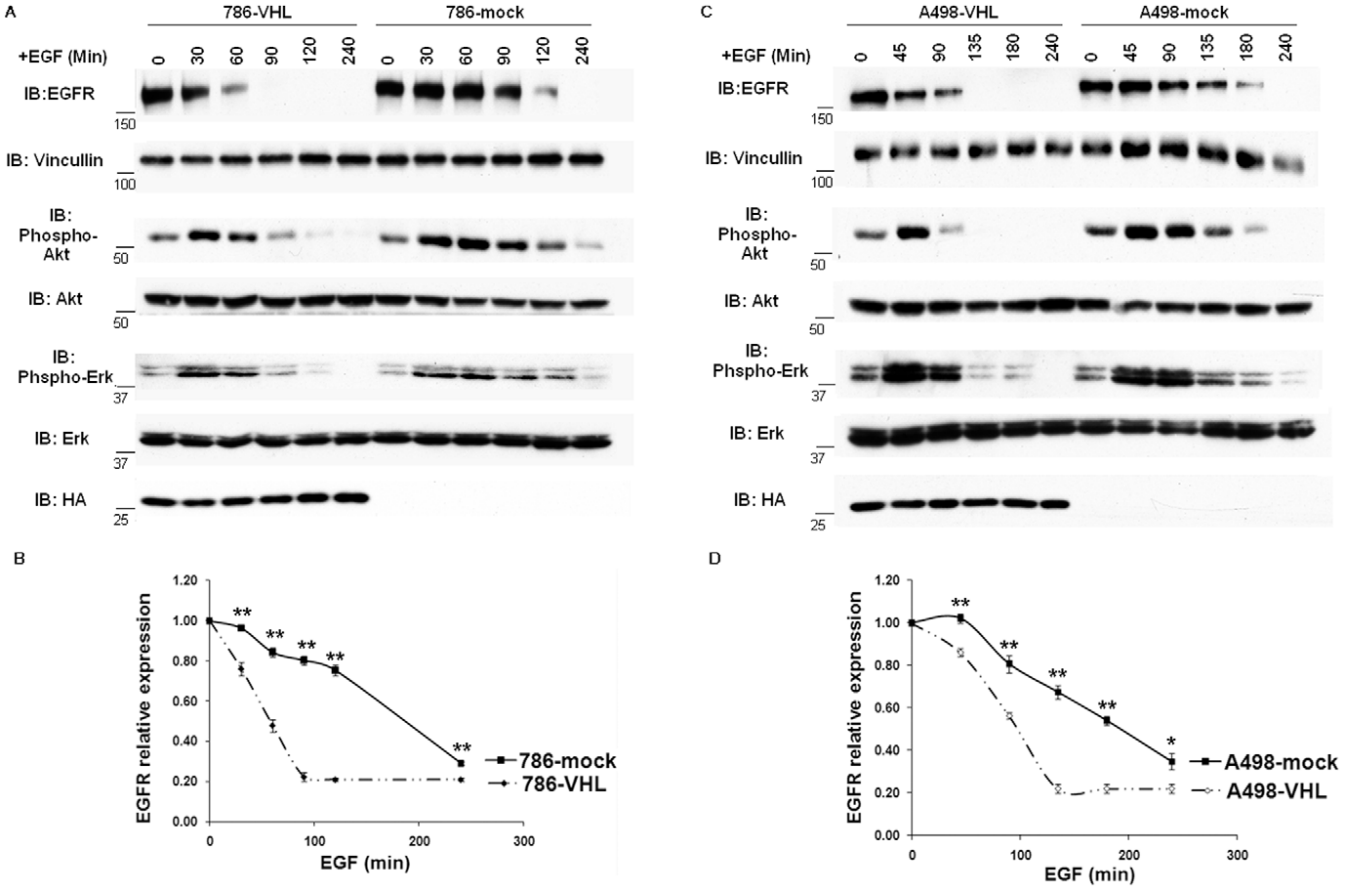
Activated EGFR had higher stability in VHL-deficient ccRCC cells than in VHL-expressing ccRCC cells. A. Renal carcinoma 786-O cells transfected to produce wild type HA-VHL (786-VHL) or with an empty plasmid (786-mock) were starved for two hours in serum free DMEM media before addition of 30 ng/ml EGF. Total cell lysates were prepared at indicated time points and immunoblotted with the indicated antibodies. Anti-HA blot detected HA-VHL. B. The EGFR signals in Fig. 1A were normalized over Vinculin via densitometry and plotted over time. Means and SDs of three separate experiments were shown. * *P*<0.05; ** *P*< = 0.001. C. The same experiment in Fig. 1A was repeated with human renal carcinoma A498 cell lines with or without HA-VHL. D. Graphic representation of Fig. 1C. * *P*<0.05; ** *P*< = 0.001.

Ubiquitylation is essential for the down-regulation of activated EGFR [30], [35]. In cells depleted of c-Cbl, activated EGFR is still ubiquitylated and degraded, albeit at a lower rate than in control cells [37], suggesting that other E3 ubiquitin ligases contributes to EGFR ubiquitylation and down-regulation. As pVHL is part of an E3 ubiquitin ligase complex that poly-ubiquitylates its targets and promotes their degradation, we explored whether pVHL participates in EGFR turnover. We compared the stabilities of activated EGFR in isogenic 786-O ccRCC cells either reconstituted with VHL (786-VHL) or expressing the empty plasmid (786-mock). The EGFR half-life was about 1 hour (h) in 786-VHL cells but approximately 3 h in 786-mock cells (Fig. 1A). The EGFR half-lives were determined by how long it took for activated EGFR to reach 50% of its starting level. As an indicator of prolonged EGFR activation, both the phospho-Akt and the phospho-Erk signals lasted longer in 786-mock cells than in 786-VHL cells after EGF stimulation (Fig. 1A).

To confirm that the difference in the stabilities of the EGFR was not limited to just one pair of ccRCC cells, we examined EGFR stabilities in another isogenic pair of ccRCC cells: A498-VHL (VHL reconstituted) and A498-mock (mock transfected). We obtained essentially the same result: The EGFR half-life was about 1.6 h in A498-VHL cells but approximately 3.2 h in A498-mock cells (Fig. 1B). To eliminate the influence of gene transcription and protein translational efficiency on EGFR turnover, we performed the same comparison between VHL-expressing and VHL-deficient cells pretreated with the translational inhibitor cycloheximide. We again observed that EGFR exhibited higher stability in VHL-deficient 786-O or A498 cells than in their VHL-reconstituted counterparts (Fig. S1A and S1B). As no new EGFR was produced after translational inhibition, the longer life-time of activated EGFR VHL-deficient cells was due to enhanced protein stability in these cells compared to their VHL-expressing counterparts. While we were carrying out our studies, another group reported the same phenomenon independently [36].

### HIF was not the only factor that stabilized activated EGFR in VHL-deficient ccRCC cells

**Figure 2:**
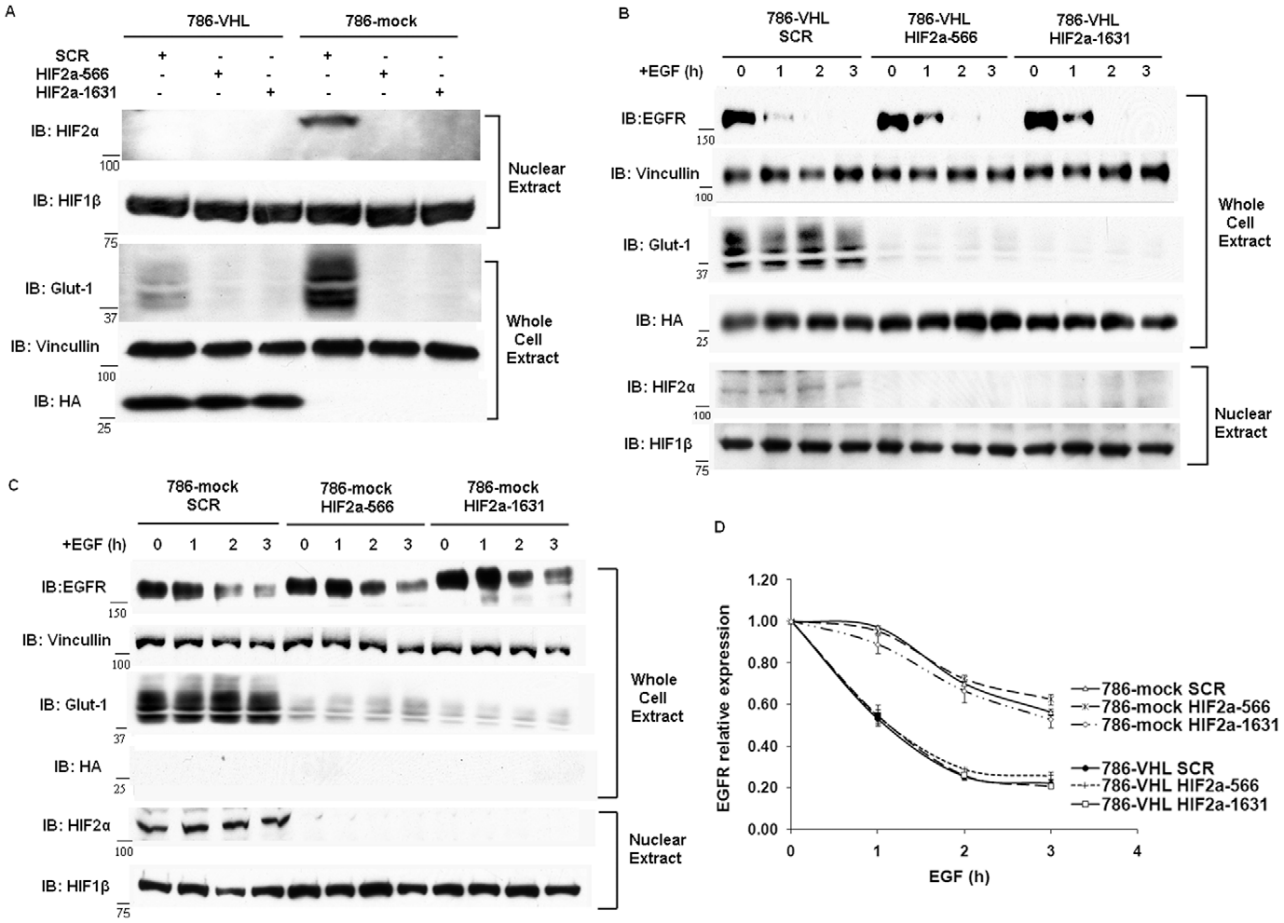
HIF was not the only factor stabilizing activated EGFR in VHL-deficient ccRCC cells. A. Western blot analysis of 786-VHL and 786-mock cells stably expressing shRNA constructs. For HIF2α and HIF1β analysis, nuclear extracts were generated and analyzed. Anti-HA blot detected HA-VHL. B. 786-VHL cells expressing SCR (control), HIF2a-566 and HIF2a-1631 (shRNA constructs against HIF2α) were treated with EGF and analyzed as described in Fig. 1A. C. 786-mock cells expressing SCR (control sequence), HIF2a-566 and HIF2a-1631 (shRNA constructs against HIF2α) were treated with EGF and analyzed as described in Fig. 2B. D. The EGFR signals in Fig. 2B and 2C were normalized over Vinculin via densitometry and plotted over time. Means and SDs of three separate experiments were shown.

786-O ccRCC cells express only HIF2α, not HIF1α [38]. Although over-expressed HIF2α increased the stability of activated EGFR in 786-VHL cells [36], it was unclear whether intrinsically higher levels of endogenous HIF2α in 786-mock cells were the major cause of stabilization of activated EGFR in these cells. To critically assess the relative contribution of endogenous HIF2α, we stably suppressed the expression of HIF2α, and as a result its target GLUT1, with two shRNA constructs (HIF2a-566 and HIF2a-1631) against HIF2α. We thereby reduced HIF2α and consequently, the HIF-target GLUT1 levels close to those observed in 786-VHL cells (Fig. 2A). The half-lives of activated EGFR were measured the same way as in Fig. 1A. HIF2α suppression in 786-VHL cells did not change the half-lives of activated EGFR (approximately 1.2 h) (Fig. 2B and 2D). Interestingly, the very successful HIF2α suppression in 786-mock cells did not significantly reduce the half-lives of activated EGFR either (Fig. 2C and 2D). The EGFR half-lives in 786-mock cells expressing either SCR or HIF2α shRNA sequences were all >3 h, and no statistically significant differences between the degradation curves were found. Consistent with this, hypoxia mimetics DFO or CoCl_2_ that successfully increased the expression of endogenous HIF2α and its target GLUT1 in 786-VHL cells also failed to significantly increase the stability of activated EGFR in these cells (Fig. S2). This suggested that in 786-mock cells endogenous HIF2α was not the only factor stabilizing activated EGFR.

### Proteasome inhibitors prevented degradation of activated EGFR in both 786-VHL and 786-mock cells, while lysosome inhibitors only stabilized activated EGFR in 786-mock cells

**Figure 3:**
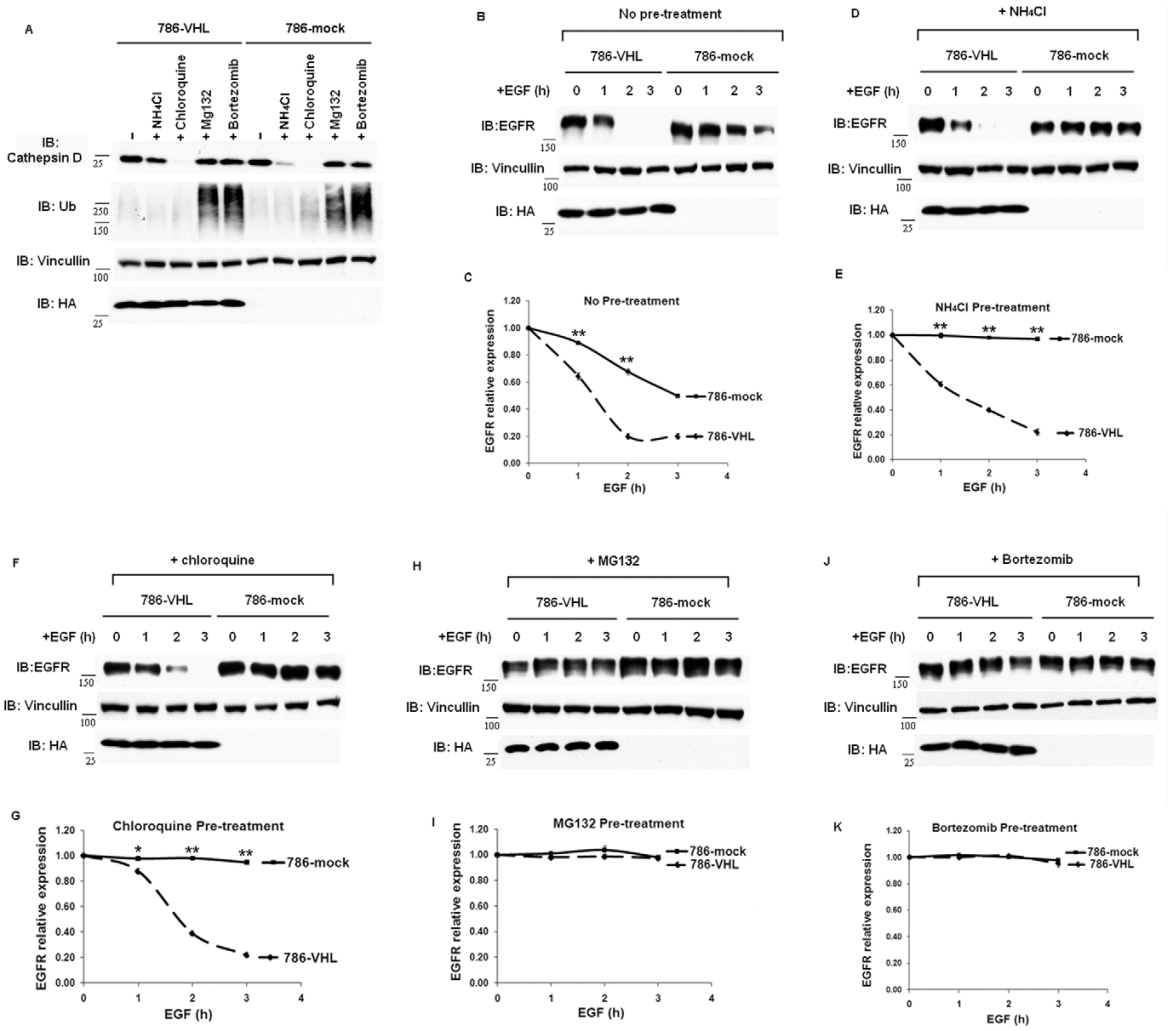
Proteasome inhibitors prevented degradation of activated EGFR in both 786-VHL and 786-mock cells, while lysosome inhibitors only stabilized activated EGFR in 786-mock cells. A. 786-VHL and 786-mock cells were treated with either lysosome inhibitors (NH_4_Cl or Chloroquine) for twenty-four hours or proteasome inhibitors (MG132 or Bortezomib) for two hours. The lysates were analyzed with indicated antibodies. Anti-HA blot detected HA-VHL. Analysis of half-lives of activated EGFR in 786-VHL and 786-mock cells: with no pretreatment (B) and its graphic representation (C), pretreated with NH_4_Cl (D) and its graphic representation (E), pretreated with Chloroquine (F) and its graphic representation (G), pretreated with MG132 (H) and its graphic representation (I), pretreated with Bortezomib (J) and its graphic representation (K). * *P*<0.05; ** *P*< = 0.001.

Poly-ubiquitylation and proteasome-mediated degradation of the activated EGFR is not a well-accepted mechanism of EGFR down-regulation [39], [40]. However, disruption of either ubiquitylation or proteasome functions by a temperature sensitive mutant of the ubiquitin activating enzyme E1 or by inhibitors has been shown to block degradation of activated EGFR to some degree [37], [41]. We investigated whether lysosomal and proteasomal degradation regulated the stabilities of the activated EGFR in 786-VHL and 786-mock cells by pretreating the cells with the lysosome inhibitors NH_4_Cl or chloroquine, or proteasome inhibitors MG132 or Bortezomib. Twenty-four hours pretreatment of the cells with NH_4_Cl slightly reduced the mature form of Cathepsin D, a lysosomal aspartyl protease, in both 786-VHL and 786-mock cells, and chloroquine had a much greater effect (Fig. 3A). This suggested that the lysosome was successfully inhibited [42]. In contrast, pretreatment with MG132 or Bortezomib for two hours did not decrease the levels of the mature form of Cathepsin D. Instead they significantly increased the poly-ubiquitylated signals in the lysates, showing that the proteasome function was blocked so the ubiquitylated protein could not be degraded (Fig. 3A). Surprisingly, neither of the lysosome inhibitors significantly increased the half-lives of the activated EGFR in the 786-VHL cells: No pretreatment: 1.3 h; NH_4_Cl, 1.5 h; chloroquine, 1.7h (Fig. 3B, 3C, 3D, 3E, 3F, and 3G). Instead, both lysosome inhibitors significantly stabilized the activated EGFR in VHL-deficient cells; The half-life of activated EGFR in untreated 786-mock was 3 h, while in NH_4_Cl- or chloroquine- treated 786-mock cells the activated EGFR was not degraded during the experiment (Fig. 3B, 3C, 3D, 3E, 3F, and 3G). The pretreament of the ccRCC cells with proteasome inhibitors, however, abolished the stability differences of the activated EGFR between the 786-VHL and 786-mock cells: the activated EGFR was not degraded during the experiment (Fig. 3B, 3C, 3H, 3I, 3J, and 3K). This suggested that the faster turnover of activated EGFR in VHL-expressing ccRCC cells was more dependent on proteasome than on lysosome, and both proteasome and lysosome were important in degrading activated EGFR in VHL-deficient ccRCC cells.

### c-Cbl suppression only significantly stabilized activated EGFR in VHL-deficient cells but not in VHL-expressing cells

**Figure 4:**
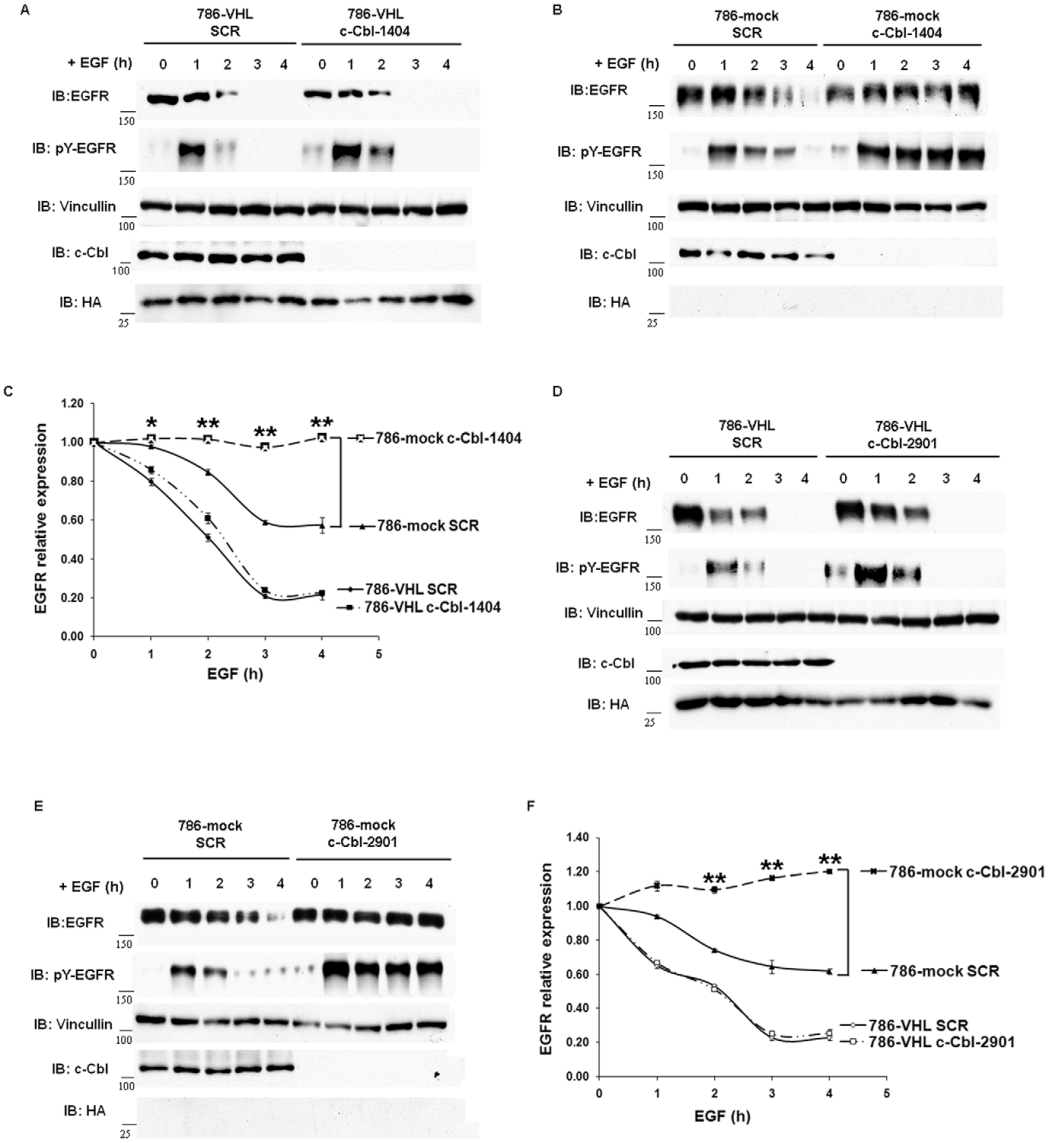
c-Cbl suppression only significantly stabilized activated EGFR in VHL-deficient cells but not in VHL-expressing cells. A. 786-VHL cells stably expressing either a control shRNA sequence (SCR) or c-Cbl-1404 were treated with EGF and analyzed with indicated antibodies as described in Figure 1A. Anti-pY-EGFR detected phospho-tyrosine 1086 on EGFR. Anti-HA blot detected HA-VHL. B. 786-mock cells stably expressing either SCR or c-Cbl-1404 were treated with EGF and analyzed with indicated antibodies as described in Figure 4A. C. The EGFR signals in Fig. 4A and 4B were normalized over Vinculin signals via densitometry and plotted over time. Means and SDs of three separate experiments were shown. * *P*<0.05; ** *P*< = 0.001. D. 786-VHL cells stably expressing either SCR or c-Cbl-2901 were treated with EGF and analyzed with indicated antibodies as described in Figure 4A. E. 786-mock cells stably expressing either SCR or c-Cbl-2901 were treated with EGF and analyzed with indicated antibodies as described in Figure 4A. F. Graphic representation of Fig. 4D and 4E. * *P*<0.05; ** *P*< = 0.001.

c-Cbl is the major E3 ubiquitin ligase to target activated EGFR. C-Cbl binds to tyrosyl phosphorylated EGFR and mono-ubiquitylates the receptor, leading to endocytosis and sorting of EGFR towards its lysosome-mediated degradation [26], [29], [33], [37]. We investigated whether the higher EGFR turnover rate in 786-VHL cells was mostly due to increased c-Cbl activity toward EGFR in these cells. To test this, we infected 786-VHL cells with shRNA constructs expressing either a control sequence (SCR) or c-Cbl-1404, which successfully down-regulated the expression of c-Cbl (Fig. 4A). After drug selection of polyclonal cells stably expressing these constructs, we compared the half-lives of activated EGFR in these cells. If elevated c-Cbl activity in 786-VHL cells was mostly responsible for the enhanced turnover of activated EGFR, then depletion of c-Cbl in these cells should prolong the half-life of activated EGFR to that of 786-mock cells. We observed, however, the opposite: loss of c-Cbl did not significantly change the 2 h half-life of activated EGFR in 786-VHL cells (Fig. 4A and 4C). Western blotting with an antibody that detected EGFR phosphorylated on tyrosine 1068 did show that loss of c-Cbl very moderately increased the overall levels of active EGFR before and after EGF stimulation (Fig. 4A). In VHL-deficient 786-mock cells, however, c-Cbl suppression essentially prevented the degradation of the already more stable EGFR: 786-mock SCR, EGFR half-life >4 h; 786-mock c-Cbl-1404, the activated EGFR was not degraded during the experiment (Fig. 4B and 4C). After EGF stimulation, neither the EGFR nor the phospho-EGFR levels decreased in VHL-deficient cells during the experiment (Fig. 4B). This suggested that c-Cbl hyperactivity was unlikely the reason that activated EGFR was degraded faster in VHL-expressing cells, and c-Cbl and pVHL collaborated to down-regulate the activated EGFR. To ensure that the results were not caused by off-target effects of c-Cbl-1404, we repeated the experiment with another construct, c-Cbl-2901, which was equally effective against c-Cbl. We obtained very similar results (Fig. 4D, 4E, and 4F), suggesting that the off-target effects of shRNA were not the cause of the observed results.

### pVHL promoted c-Cbl-independent poly-ubiquitylation of the activated EGFR

**Figure 5:**
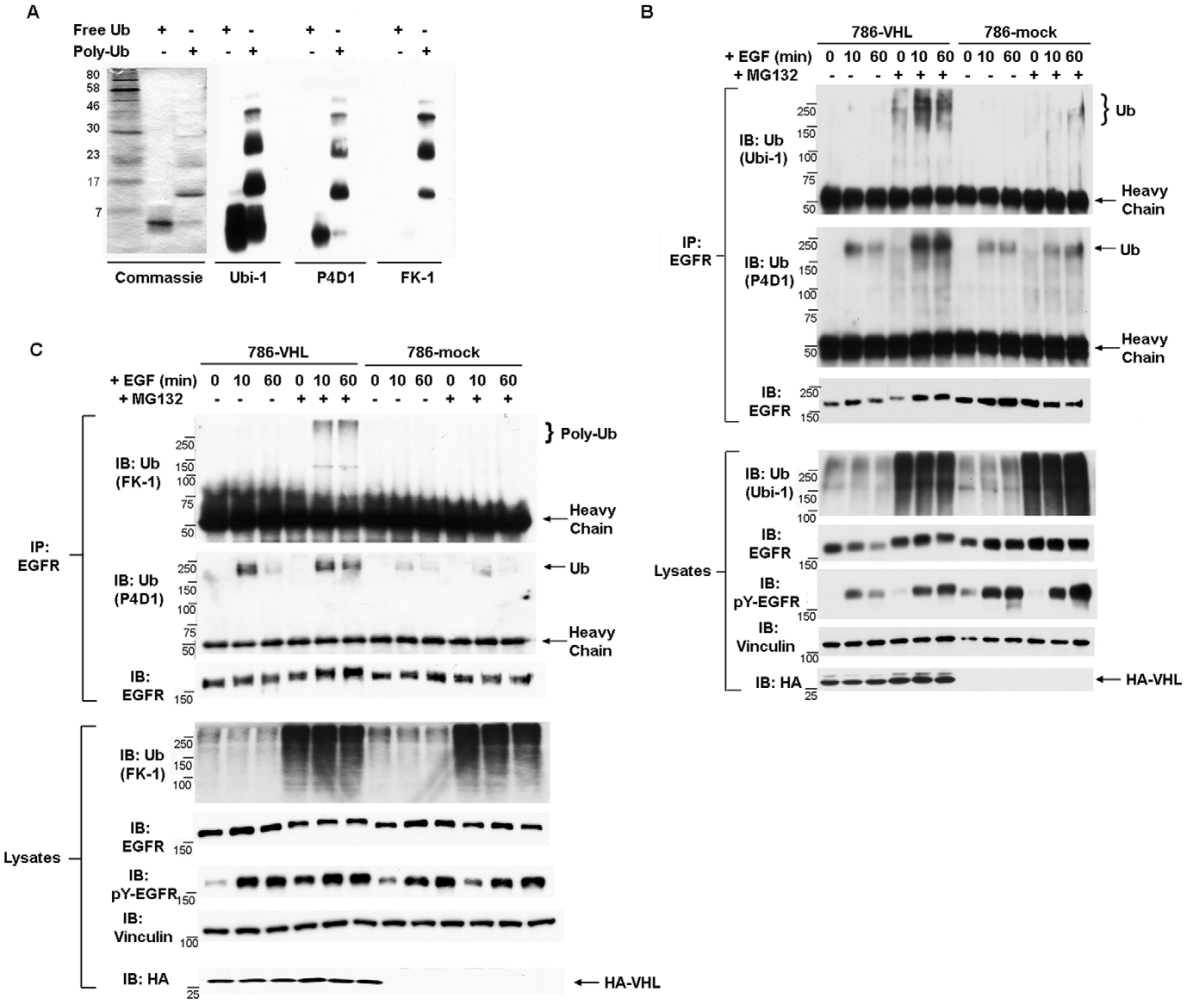
pVHL promoted poly-ubiquitylation of the activated EGFR. A. 1 µg each of free ubiquitin and Poly-ubiquitin were resolved by SDS-PAGE and detected by either commassie stain or indicated anti-Ub antibodies. B. 786-VHL and 786-mock cells were treated with EGF and were starved of serum for two hours in the presence of DMSO (the solvent) or 10 µM MG132 before the addition of 30 ng/ml EGF. The lysates were prepared with EBC buffer at indicated time and immunoprecipitated with anti-EGFR antibody. The immunoprecipitates were blotted with anti-Ub (Ubi), anti-Ub (P4D1), and anti-EGFR sequentially with membrane stripping between blots. The lysates were blotted with indicated antibodies. C. 786-VHL and 786-mock cells were treated with EGF and were starved of serum for two hours in the presence of DMSO (the solvent) or 10 µM MG132 before the addition of 30 ng/ml EGF. Denaturing IP was performed with anti-EGFR antibody with these lysates. The immunoprecipitates were blotted with anti-Ub (FK-1), anti-Ub (P4D1), and anti-EGFR sequentially with membrane stripping between blots. The lysates were blotted with indicated antibodies.

As stated before, it is controversial as to whether activated EGFR is poly-ubiquitylated upon EGF stimulation [29], [33], [34], [35]. In previous publications, ubiquitylation of activated EGFR was analyzed in the absence of proteasome inhibitor. As proteins conjugated with poly-ubiquitin chains except lys-63-linked chain were quickly cleared by the proteasome [43], we hypothesized that the poly-ubiquitylated EGFR signals may be more readily visualized if proteasome function was disabled. To investigate whether pVHL promotes the poly-ubiquitylation of activated EGFR, we first tested the specificities of anti-ubiquitin (Ub) antibodies against free Ub and Poly-Ub with western blots. As reported, anti-Ub (Ubi-1) and anti-Ub (P4D1) recognized both free Ub and Poly-Ub, while anti-Ub (FK-1) only detected Poly-Ub (Fig. 5A) [29]. We then treated 786-VHL and 786-mock cells with DMSO or MG132 for two hours before EGF stimulation. The lysates were then immunoprecipitated with anti-EGFR antibody before blotting with an anti-Ub antibody (Ubi-1). Activated EGFR associated with diffusive high molecular weight Ub signals in MG132-treated VHL-expressing cells, but not in DMSO treated VHL-expressing cells or in DMSO or MG132 treated VHL-deficient cells (Fig. 5B, first panel). Reprobing with another anti-Ub antibody (P4D1) revealed that Ubi1-specific Ub signals were quite different from the P4D1-specific Ub signals (Fig. 5B, first and second panels). The P4D1-specifc Ub signals were present near 250KDa molecular weight marker and were more focused, and they were present in all the EGF stimulated samples. Furthermore, the P4D1-specific Ub signals were enhanced by MG132 treatment in VHL-expressing cells but not in VHL-deficient cells (Fig. 5B, second panel). The anti-Ub blot of the lysates indicated that the overall levels of ubiquitylation were not reduced in VHL-deficient cells, so this did not cause the lack of Ubi-1-specific signals associating with activated EGFR in these cells (Fig. 5B, fourth panel). Our results suggested that pVHL promoted the ubiquitylation of the activated EGFR.

Although only after the proteasome was inhibited the activated EGFR associated with Ubi-1-specific Ub signals in VHL-expressing cells, it was possible that the ubiquitylation occurred on another protein that was tightly associated with EGFR rather than on EGFR itself. If this were the case, boiling lysates with SDS before EGFR immunoprecipitation should abolish the associated Ub signals from EGFR. Indeed, this harsh treatment stripped EGFR of non-covalently associated proteins such as c-Cbl (Fig. S3A). Silver stain of immunoprecipitated EGFR under non-denaturing and denaturing conditions revealed that denaturing IP recovered mostly the antibody and EGFR itself but removed the associating proteins (Fig. S3B). Anti-Ub (Ubi-1) and anti-Poly-Ub (FK-1) blots of immunoprecipitated EGFR under non-denaturing and denaturing conditions showed that under both conditions, the Ub signals associated with activated EGFR were much stronger in VHL-expressing cells than that in VHL-deficient cells (Fig. S4A and S4B). This suggested that the Ub signals were very tightly, possibly covalently, linked to the activated EGFR in these cells.

**Figure 6:**
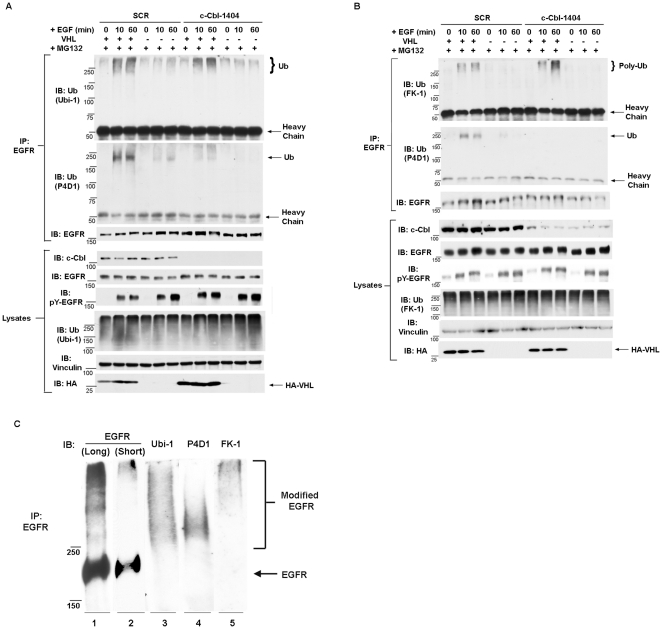
pVHL-dependent ubiquitylation of activated EGFR was c-Cbl-independent. A. 786-VHL and 786-mock cells expressing either a control shRNA sequence (SCR) or c-Cbl-1404 were starved of serum for two hours in the presence of 10 µM MG132 before the addition of 30 ng/ml EGF. The lysates were prepared with EBC buffer at indicated time and immunoprecipitated with anti-EGFR antibody. The immunoprecipitates were blotted with anti-Ub (Ubi-1), anti-Ub (P4D1), and anti-EGFR sequentially with membrane stripping between blots. The lysates were blotted with indicated antibodies. B. 786-VHL and 786-mock cells expressing either a scramble shRNA sequence (SCR) or c-Cbl-1404 were starved of serum for two hours in the presence of 10 µM MG132 before the addition of 30 ng/ml EGF. Denaturing IP was performed with anti-EGFR antibody with these lysates. The immunoprecipitates were blotted with anti-Poly-Ub (FK-1), anti-Ub (P4D1), and anti-EGFR sequentially with membrane stripping between blots. The lysates were blotted with indicated antibodies. C. 786-VHL cells were treated with MG132 and stimulated with EGF for 60 minutes. Denaturing IP with anti-EGFR antibody was performed with the lysate. The immunoprecipitated material was split and run on SDS-PAGE gel for extended time and blotted with indicated antibodies. Long: long exposure. Short: short exposure.

Because Ubi-1 and P4D1 were not specific to Poly-Ub, and EGFR was reported to be both mono-ubiquitylated and poly-ubiquitylated, it was not known whether pVHL promoted the poly-ubiquitylation of the activated EGFR. To address this, EGFR was immunoprecipitated from 786-VHL and 786-mock cells treated with DMSO or MG132 before EGF stimulation under denaturing condition. Anti-Poly-Ub (FK-1) blot revealed that Poly-Ub signals only associated with activated EGFR in VHL-expressing cells but not in VHL-deficient cells, and only after the proteasome function was inhibited (Fig. 5C).

c-Cbl is the major E3 ubiquitin ligase for activated EGFR and it was not known whether the pVHL promoted ubiquitylation of EGFR was c-Cbl-dependent. To address this, c-Cbl expression in 786-VHL and 786-mock cells were suppressed by stable expression of c-Cbl-1404 shRNA construct. Cells expressing SCR or c-Cbl-1404 were stimulated with EGF, then the lysates were immunoprecipitated with anti-EGFR antibody. Anti-Ub (P4D1) blot revealed that EGFR associated P4D1-specific Ub signals disappeared after c-Cbl suppression in both 786-VHL and 786-mock cells (Fig. 6A and 6B, second panels), suggesting that c-Cbl depletion did impact EGFR ubiquitylation. However, the high molecular weight Ub signals associated with activated EGFR in VHL-expressing cells persisted after c-Cbl depletion (Fig. 6A, first panel), suggesting that pVHL-promoted EGFR ubiquitylation was c-Cbl-independent. Repeating the same experiment with anti-Poly-Ub (FK-1) with denaturing IP confirmed the above conclusion (Fig. 6B).

Because the anti-Ub (P4D1) signals associated with EGFR was c-Cbl-dependent and were more focused near 250 KDa, and c-Cbl depletion seemed to remove the bottom half of Ubi-1-specific Ub signals (Fig. 6A, first panel), we investigated whether these anti-Ub antibodies detected different populations of Ub signals. Activated EGFR from VHL-expressing cells was immunoprecipitated under denaturing condition and run on a gel for a long time for better resolution. Ubi-1-specific Ub signal mostly overlapped with modified EGFR (Fig. 6C). P4D1-specific Ub signal overlapped with the bottom half of Ubi-1-specific Ub signal, while the Poly-Ub (FK-1) signal overlapped with the top half of Ubi-1-specific Ub signals. There was little overlap between P4D1-specific Ub signal and FK-1 Poly-Ub signal (Fig. 6C). This suggested that while Ubi-1 was detecting all forms of ubiquitin, P4D1 and FK-1 had high affinity to different sub-populations of ubiquitin.

## Discussion

*VHL* inactivation is a causal factor for the development of the ccRCC tumors. Loss of pVHL functions leads to constant activation of the HIF transcriptional factor. Blockage of tumor angiogenesis, one of the consequences of HIF activation, produces positive clinical outcomes [18]. VHL loss also causes abnormal activation of EGFR, a receptor tyrosine kinase whose uncurbed activity is oncogenic in many types of cancers.

After VHL loss, activated HIF up-regulates the expression of the EGFR agonist TGF-α [19] and enhances the translational efficiency of EGFR [20] to promote autonomous growth of *VHL*-defective ccRCC cells. Recently it was reported that degradation of activated EGFR was impaired in *VHL*-defective ccRCC cells, so that EGFR was left to promote proliferation and block apoptosis much longer to enhance oncogenesis [36]. We independently discovered the stabilization of activated EGFR in *VHL*-defective ccRCC cells and wished to critically examine the contribution of HIF and lysosome to pVHL-mediated EGFR degradation. Wang et al found that over-expression of HIF2α in VHL-expressing cells stabilized activated EGFR, which we also observed. They also showed that either VHL suppression, or over-expression of HIF2α, or hypoxia clearly delayed Rab5-mediated endosome fusion. So without VHL, HIF2α accumulated and repressed rabaptin-5 expression, and this led to delayed endosome fusion and subsequently slower lysosome-mediated turnover of activated EGFR. However, this mechanism would predict that suppression of endogenous HIF2α in 786-mock cells would restore the half-life of activated EGFR to that of 786-VHL cells. As this was missing in their paper, we performed this experiment and found that depletion of endogenous HIF2α in 786-mock cells did not significantly reduce the half-lives of activated EGFR (Fig. 2C and 2D). Furthermore, hypoxia mimetics that blocked proline hydroxylases to induce endogenous HIF2α did not significantly enhance EGFR half-lives either in VHL-expressing cells. Finally, if impaired lysosome function was the major cause of increased half-life of activated EGFR in VHL-deficient cells, then blocking lysosome function in VHL-expressing cells should prolong the EGFR half-life to the similar level as seen in VHL-deficient cells. That was not what we observed (Fig. 3). Thus we concluded that HIF was not the only factor stabilizing activated EGFR in VHL-deficient cells.

Although lysosome inhibitors did not significantly stabilize the activated EGFR in 786-VHL cells, they did further stabilize the activated EGFR in VHL-deficient cells (Fig. 3). The proteasomal inhibitors, on the other hand, blocked the degradation of activated EGFR in both VHL-expressing and VHL-deficient cells (Fig. 3). The evidence suggested that the lysosome function was critical for degradation of activated EGFR in VHL-deficient cells, and the increased proteasome-mediated degradation was the major reason that activated EGFR had a shorter half-life in VHL-expressing ccRCC cells.

Since c-Cbl is the major E3 that ubiquitylates the activated EGFR, which leads to its lysosome-mediated destruction, we studied the contribution of c-Cbl to the EGFR turnover in ccRCC cells. Suppression of c-Cbl expression did not significantly increase the stabilities of the activated EGFR in VHL-expressing cells. However, c-Cbl loss made the activated EGFR very stable in VHL-deficient cells (Fig. 4). As the effects of c-Cbl suppression on EGFR stability in ccRCC cells were very similar to that of lysosome inhibitors, this was consistent with the notion that c-Cbl-mediated ubiquitylation of EGFR led to lysosome-mediated degradation. Furthermore, c-Cbl collaborated with pVHL to promote the degradation of activated EGFR. Without both, EGFR was activated but remained stable.

It is controversial as to whether activated EGFR is poly-ubiquitylated and whether poly-ubiquitylated EGFR is subjected to proteasomal or lysosomal degradation. We suspected that pVHL may promote poly-ubiquitylation of activated EGFR. We indeed observed VHL-dependent poly-ubiquitylation of activated EGFR but only when proteasome was inhibited (Fig. 5C), suggesting that activated EGFR was quickly turned over by the proteasome under normal conditions. The comparison of non-denaturing IP and denaturing IP suggested that the VHL-dependent Poly-ub was tightly, possibly covalently, linked to activated EGFR (Fig. S4B). Interestingly, this VHL-dependent poly-ubiquitylation of activated EGFR was c-Cbl-independent (Fig. 6A and 6B). As EGFR-associated P4D1-specific Ub signals were c-Cbl-dependent (Fig. 6A and 6B), c-Cbl was reported to mainly mono-ubiquitylate activated EGFR [29], [33], and P4D1-specific anti-Ub signal was below the Poly-Ub (FK-1) signal and overlapped with the bottom of the Ubi-1-specific Ub signal (Fig. 6C), it was possible that P4D1 was mainly detecting mono-ubiquitylated EGFR. This certainly deserves further investigation. In all, our evidence suggested that pVHL promoted poly-ubiquitylation on the activated EGFR which likely led to proteasome-mediated degradation.

Because we observed that (1) c-Cbl promoted P4D1-specific Ub signals on activated EGFR; (2) VHL-dependent poly-ubiquitylation on EGFR was c-Cbl independent; (3) c-Cbl loss and lysosome inhibition had very similar effects on EGFR stability in ccRCC cells; (4) proteasome inhibitors, not lysosome inhibitors, abolished the EGFR stability differences in VHL-expressing and VHL-deficient cells, it was likely that pVHL-containing E3 complex and c-Cbl promoted different kinds of ubiquitylation on activated EGFR. Further functional and biochemical investigation will help to resolve this issue.

In total, our results suggest that a pVHL-dependent poly-ubiquitylation and proteasomal degradation pathway plays a very important role in suppression of EGFR activity through degradation of activated EGFR. It will be interesting to investigate whether similar pVHL-dependent regulation of EGFR occurs in other cell types. It will also be interesting to learn whether pVHL directly binds to and promotes ubiquitylation of activated EGFR and what the binding signal is, and what type of ubiquitin chain pVHL-containing E3 ubiquitin ligase complex is adding onto EGFR. As disruption of ubiquitylation [37], blocking of proteasome functions by inhibitors [41], or VHL loss [36] all resulted in EGFR accumulating at early stages during endocytosis which led to the conclusion that these events blocked the proper endosome transportation/sorting, it is worth investigating how and where these trapped EGFR proteins were eventually degraded by proteasome.

## Materials and Methods

### Cell culture

ccRCC cells such as 786-O and A498 with or without pCDNA3 based wild type HA-VHL were described previously [17]. After transfection, the cells were treated with 1 mg/ml neomycin to kill the cells that do not stably express the plasmids. After three passages all the cells became drug-resistant. The VHL status and the HIF activity of the cell lines were confirmed by anti-HA and anti-GLUT1 immunoblots. Cell lines were maintained in glutamine-containing DMEM medium with 10% Fetal Bovine Serum (FBS) plus 1% penicillin and streptomycin. For Epidermal Growth Factor (EGF) stimulation, sub-confluent cells with similar densities were washed and starved in DMEM medium for two hours before addition of EGF (Biovision, Mountainview, CA) to 30 ng/ml final concentration. At indicated time points, the cells were washed with PBS and lysed with EBC buffer (50 mM Tris (pH 8), 120 mM NaCl, 0.5% NP-40) containing protease inhibitor cocktail (Roche complete, 11836153001) and phosphatase inhibitor cocktail (final concentrations: 5 mM sodium Fluoride, 1 mM sodium orthovanadate, 1 mM sodium pyrophosphate, and 1 mM β-glycerophosphate).

For hypoxia mimetic treatment, 100 µM Deferoxamine (DFO, an iron chelator that depletes iron at the active site of the prolyl hydroxylases) or 100 µM Cobalt Chloride (CoCl_2_, which replaces iron at the active site of the prolyl hydroxylases) was added to the cell culture media for twenty-two hours before the cells were starved and treated with EGF in the presence of the chemicals. For cycloheximide (CHX) treatment, 70–80% confluent cells were treated with CHX at 100 µg/ml for two hours while the cells were starved and treated with EGF in the presence of CHX.

### Analysis of EGFR stability in the absence or the presence of lysosomal inhibitors or proteasome inhibitor

To inhibit the lysosomal function, lysosome inhibitors were added to the 786-O cells with or without stably expressed HA-VHL and incubated for twenty-two hours. Then the cells were starved of FBS for two hours in the presence of lysosome inhibitors before the addition of 30 ng/ml EGF. Final concentrations of inhibitors: 10 mM NH_4_Cl (Amresco, Solon, OH); 100 µM Chloroquine (Sigma, Saint Louis, MO). To inhibit the proteasomal function, the same cells were starved of FBS for two hours in the presence of 10 µM MG132 (Cayman Chemical, Ann Arbor, MI) or 50 nM Bortezomib (S1013) (Sellick Chemicals, Houston, TX) before two-hour starvation and the addition of EGF in the presence of the inhibitors.

### Western Blot analysis

Total cellular lysates were prepared with EBC buffer (50 mM Tris (pH 8), 120 mM NaCl, 0.5% NP-40). The protein concentrations of the lysates were determined by a protein assay kit (500-0006) from Bio-rad (Hercules, CA). NE-PER Nuclear and Cytoplasmic Extraction Kit (Thermo Scientific, Rockford, IL, Cat# 78833) was used to fractionate the cells according to manufacturer's instruction. In brief, the cells were trypsinized and precipitated before addition of reagents CERI and CERII to extract the cytoplasmic proteins. The remaining pellets were further added NER reagent and vortexed to extract the nuclear proteins. The same amount of total protein from each sample was boiled with sample buffer before being resolved by SDS-PAGE and analyzed with standard western blot techniques. The blots were developed with Super Signal Pico substrate (Pierce Biotechnology, Rockford, IL) or Immobilon Western substrate (Millipore, Billerica, MA). Antibodies against EGFR (A300-387A, A300-388A) are from Bethyl Laboratories (Montgomery, TX). Antibodies against phospho-EGFR (Tyr1086) (3777), Phospho-Akt (Thr308) (2965), Akt (9272), Phospho-Erk (1/2) (Thr202/Tyr204) (9101), Erk (1/2) (9102) were from Cell Signaling Technology (Boston, MA). Antibodies against HIF2α (NB100-480), HIF1β (NB100-124), GLUT1 (NB300-666) were purchased from Novus Biologicals (Littleton, CO). Antibodies against Cathepsin D (sc-6486), PARP1 (sc-7150), anti-HA-epitope (sc-7392), anti-c-Cbl (sc-170), anti-Ub (P4D1) (sc-8017), and Vinculin (sc-73614) were from Santa Cruz Biotechnology (Santa Cruz, CA). Anti-Ub antibody (Ubi-1) (Mab1510) was from Millipore (Billerica, MA), and anti-Poly-Ub antibody (FK1) (D071-3) was from MBL (Nagoya, Japan). For sequential western blots, the membranes were stripped with gentle review buffer (Amresco, Solon, OH) between blots. The intensity of a band was determined with NIH imageJ software (Wayne Rasband, National Institutes of Health). Free Ub (BML-UW8795) was obtained from Enzo Life Sciences (Plymouth meeting, PA), and Poly-Ub (Ub1-7, K48-linked) (UC-240) was from Boston Biochem (Cambridge, MA).

For silver stain, the proteins were resolved by SDS-PAGE. Then the gel was fixed with a mixture of 50% methanol and 5% acetic acid. Then it was washed sequentially with 50% methanol and water for ten minutes each. The gel was sensitized with 0.02% sodium thiosulfate for one minute, rinsed twice with water, then incubated with 0.1% silver nitrate at 4°C for twenty minutes. The gel was rinsed twice with water, then developed with 0.04% formalin in 2% sodium carbonate until the desired result was achieved. The reaction was stopped by 5% acetic acid.

### Short Hairpin RNAs (shRNAs)

shRNA constructs in E. coli glyceryl stock were obtained from Sigma (Saint Louis, MO). The sequences were: SCR: GCGCGCUUUGUAGGAUUCGTT; HIF2a-1631: CGACCTGAAGATTGAAGTGAT; HIF2a-566: CCATGAGGAGATTCGTGAGAA; c-Cbl-1401: TCGCAGAGAAATCGGGCATTT; c-Cbl-2901: GAGAAGTCAGATGGTTTATTT.

The DNA was purified with Maxi-prep kit (Qiagen, Valencia, CA). Lentiviral plasmids were transfected into the Phoenix packaging cell line using Lipofectamine 2000 (Invitrogen, Carlsbad, CA) according to the manufacturer's instructions. Tissue culture medium was collected 48 h after transfection, passed through a 0.45-µm-pore-size filter, and added to cells in the presence of 8 µg/ml polybrene. Two days later 1 µg/ml puromycin was added to kill the cells that do not stably express the plasmids. After three passages in the presence of puromycin all the cells became drug-resistant. The efficacy of the shRNA suppression was confirmed by western blot and/or real-time PCR before the cells were used for experiments.

### Denaturing and non-denaturing immunoprecipitation

786-O cells with or without stably expressed HA-VHL at about 80% confluence were starved with FBS-free medium containing 10 µM MG132 for two hours, then 30 ng/ml EGF was added and the cells were harvested at the following time points: 0, 10 minutes, 60 minutes. The cells were lysed with EBC buffer containing protease inhibitor cocktail and phosphatase inhibitor cocktail on ice. The lysates were sonicated for 15 seconds before centrifugation as 15,000 rpm for 10 minutes. The cleared supernatants were transferred to new eppendorf tubes. After measurement of protein concentrations, 3 mg total protein from each sample were used for both non-denaturing IP and denaturing IP. For denaturing IP, 1.5 mg total protein was mixed with equal volume of 1% SDS solution to reach a final concentration of 0.5% SDS. The mixtures were heated for 5 minutes at 100°C. EBC buffer containing protease inhibitor cocktail and phosphatase inhibitor cocktail was added to dilute the SDS to 0.1% final concentration. Samples for non-denaturing IP were not boiled with SDS before immunoprecipitation. To each sample, 1 µg anti-EGFR antibody was added and the tubes were rotated at 4°C overnight. The mixtures were centrifuged at 15,000 rpm for 10 minutes. The cleared lysates were transferred to new tubes, and 20 µl protein A beads were added to each sample. The mixtures were rotated for one hour at 4°C before centrifugation at 7000 rpm for thirty seconds. The beads were washed with NETN buffer (50 mM Tris-HCl pH 7.9, 100 mM NaCl, 1 mM EDTA, 10% glycerol, 0.2% NP-40) for three times. 15 µl 2× protein loading buffer were added to each tube. The samples were boiled for five minutes at 100°C. The proteins were resolved with SDS-PAGE gels before western blot analysis.

### Statistical analysis

The western blot signals of EGFR and Vinculin were quantified with NIH imageJ software. The EGFR/Vinculin ratios from three different experiments were calculated and plotted over time as means ± standard deviation (SD). The ratios at t = 0 were artificially set as 1. Differences between the means of any two samples at a given time of EGF treatment were evaluated by the Unpaired t-test using the SigmaPlot program. *P* values <0.05 were considered statistically significant. *: *P*<0.05; **: *P*< = 0.001.

## Supporting Information

Figure S1 **Activated EGFR had higher stability in VHL-deficient ccRCC cells than in VHL-expressing ccRCC cells in the presence of cycloheximide.** A. Renal carcinoma 786-O cells transfected to produce wild type HA-VHL (786-VHL) or with an empty plasmid (786-mock) were starved for two hours in serum free DMEM media in the presence of 100 µg/ml cycloheximide (CHX) before addition of 30 ng/ml EGF. Total cell lysates were prepared at indicated time points and immunoblotted with the indicated antibodies. B. The same experiment in Fig. S1A was repeated with human renal carcinoma A498 cell lines with or without HA-VHL.
(TIF)

Figure S2 **Hypoxia mimetics did not significantly increase the half-lives of activated EGFR in VHL-expressing ccRCC cells.** 786-VHL (A) and 786-mock (B) cells were either untreated or treated with 100 µM hypoxia mimetics (DFO or CoCl_2_) for twenty-two hours. Then the cells were starved of serum for two hours in the absence or the presence of chemicals before the addition of 30 ng/ml EGF. The lysates were prepared with EBC buffer at indicated time and immunoblotted with indicated antibodies. Anti-HA blots detected HA-VHL.
(TIF)

Figure S3 **Denaturing IP removed proteins associated with activated EGFR.** A. 786-VHL and 786-mock cells were starved and treated with 10 µM MG132 for two hours before addition of EGF. EBC lysates were generated. Half of the lystates were used for non-denaturing IP with anti-EGFR antibody, and the other half used for denaturing IP. The immunoprecipitated materials were blotted with indicated antibodies. B. A similar experiment as described in S3A was performed and the immunoprecipitated materials were resolved on a SDS-PAGE gel before silver staining.
(TIF)

Figure S4 **pVHL-dependent poly-ubiquitylation on the activated EGFR persisted after denaturing IP.** A. 786-VHL and 786-mock cells expressing c-Cbl-1404 to avoid the interference of P4D1-specific Ub signals were starved of serum for two hours in the presence of 10 µM MG132 before the addition of 30 ng/ml EGF. The lysates were prepared with EBC buffer at indicated time. Half of the lysates were used for non-denaturing IP and the other half were used for denaturing IP. The immunoprecipitates were blotted with anti-Ub (Ubi-1) and anti-EGFR sequentially with membrane stripping between blots. The lysates were blotted with indicated antibodies. B. The same experiment as in S4A was performed and the immunoprecipitates were blotted with anti-poly Ub (FK-1) and anti-EGFR sequentially with membrane stripping between blots. The lysates were blotted with indicated antibodies.
(TIF)

## References

[1] Linehan WM, Vasselli J, Srinivasan R, Walther MM, Merino M (2004). Genetic basis of cancer of the kidney: disease-specific approaches to therapy.. Clin Cancer Res.

[2] Kaelin WG (2002). Molecular basis of the VHL hereditary cancer syndrome.. Nat Rev Cancer.

[3] Kamura T, Koepp DM, Conrad MN, Skowyra D, Moreland RJ (1999). Rbx1, a component of the VHL tumor suppressor complex and SCF ubiquitin ligase.. Science.

[4] Ohh M, Park CW, Ivan M, Hoffman MA, Kim TY (2000). Ubiquitination of hypoxia-inducible factor requires direct binding to the beta-domain of the von Hippel-Lindau protein.. Nat Cell Biol.

[5] Yang H, Minamishima YA, Yan Q, Schlisio S, Ebert BL (2007). pVHL acts as an adaptor to promote the inhibitory phosphorylation of the NF-kappaB agonist Card9 by CK2.. Mol Cell.

[6] Thoma CR, Toso A, Gutbrodt KL, Reggi SP, Frew IJ (2009). VHL loss causes spindle misorientation and chromosome instability.. Nat Cell Biol.

[7] Schraml P, Frew IJ, Thoma CR, Boysen G, Struckmann K (2009). Sporadic clear cell renal cell carcinoma but not the papillary type is characterized by severely reduced frequency of primary cilia.. Mod Pathol.

[8] Mikhaylova O, Ignacak ML, Barankiewicz TJ, Harbaugh SV, Yi Y (2008). The von Hippel-Lindau tumor suppressor protein and Egl-9-Type proline hydroxylases regulate the large subunit of RNA polymerase II in response to oxidative stress.. Mol Cell Biol.

[9] Semenza GL (2007). Hypoxia-inducible factor 1 (HIF-1) pathway.. Sci STKE.

[10] Ivan M, Kondo K, Yang H, Kim W, Valiando J (2001). HIFalpha targeted for VHL-mediated destruction by proline hydroxylation: implications for O2 sensing.. Science.

[11] Jaakkola P, Mole DR, Tian YM, Wilson MI, Gielbert J (2001). Targeting of HIF-alpha to the von Hippel-Lindau ubiquitylation complex by O2-regulated prolyl hydroxylation.. Science.

[12] Epstein AC, Gleadle JM, McNeill LA, Hewitson KS, O'Rourke J (2001). C. elegans EGL-9 and mammalian homologs define a family of dioxygenases that regulate HIF by prolyl hydroxylation.. Cell.

[13] Ivan M, Haberberger T, Gervasi DC, Michelson KS, Gunzler V (2002). Biochemical purification and pharmacological inhibition of a mammalian prolyl hydroxylase acting on hypoxia-inducible factor.. Proc Natl Acad Sci U S A.

[14] Semenza GL (2003). Targeting HIF-1 for cancer therapy.. Nat Rev Cancer.

[15] Kondo K, Klco J, Nakamura E, Lechpammer M, Kaelin WG (2002). Inhibition of HIF is necessary for tumor suppression by the von Hippel-Lindau protein.. Cancer Cell.

[16] Zimmer M, Doucette D, Siddiqui N, Iliopoulos O (2004). Inhibition of hypoxia-inducible factor is sufficient for growth suppression of VHL-/- tumors.. Mol Cancer Res.

[17] Kondo K, Kim WY, Lechpammer M, Kaelin WG (2003). Inhibition of HIF2alpha is sufficient to suppress pVHL-defective tumor growth.. PLoS Biol.

[18] Rini BI (2005). VEGF-targeted therapy in metastatic renal cell carcinoma.. Oncologist.

[19] de Paulsen N, Brychzy A, Fournier MC, Klausner RD, Gnarra JR (2001). Role of transforming growth factor-alpha in von Hippel–Lindau (VHL)(-/-) clear cell renal carcinoma cell proliferation: a possible mechanism coupling VHL tumor suppressor inactivation and tumorigenesis.. Proc Natl Acad Sci U S A.

[20] Franovic A, Gunaratnam L, Smith K, Robert I, Patten D (2007). Translational up-regulation of the EGFR by tumor hypoxia provides a nonmutational explanation for its overexpression in human cancer.. Proc Natl Acad Sci U S A.

[21] Lee SJ, Lattouf JB, Xanthopoulos J, Linehan WM, Bottaro DP (2008). Von Hippel-Lindau tumor suppressor gene loss in renal cell carcinoma promotes oncogenic epidermal growth factor receptor signaling via Akt-1 and MEK-1.. Eur Urol.

[22] Smith K, Gunaratnam L, Morley M, Franovic A, Mekhail K (2005). Silencing of epidermal growth factor receptor suppresses hypoxia-inducible factor-2-driven VHL-/- renal cancer.. Cancer Res.

[23] Riese DJ, Gallo RM, Settleman J (2007). Mutational activation of ErbB family receptor tyrosine kinases: insights into mechanisms of signal transduction and tumorigenesis.. Bioessays.

[24] Yarden Y, Sliwkowski MX (2001). Untangling the ErbB signalling network.. Nat Rev Mol Cell Biol.

[25] Jorissen RN, Walker F, Pouliot N, Garrett TP, Ward CW (2003). Epidermal growth factor receptor: mechanisms of activation and signalling.. Exp Cell Res.

[26] Levkowitz G, Waterman H, Zamir E, Kam Z, Oved S (1998). c-Cbl/Sli-1 regulates endocytic sorting and ubiquitination of the epidermal growth factor receptor.. Genes Dev.

[27] Levkowitz G, Waterman H, Ettenberg SA, Katz M, Tsygankov AY (1999). Ubiquitin ligase activity and tyrosine phosphorylation underlie suppression of growth factor signaling by c-Cbl/Sli-1.. Mol Cell.

[28] Waterman H, Katz M, Rubin C, Shtiegman K, Lavi S (2002). A mutant EGF-receptor defective in ubiquitylation and endocytosis unveils a role for Grb2 in negative signaling.. EMBO J.

[29] Haglund K, Sigismund S, Polo S, Szymkiewicz I, Di Fiore PP (2003). Multiple monoubiquitination of RTKs is sufficient for their endocytosis and degradation.. Nat Cell Biol.

[30] Huang F, Goh LK, Sorkin A (2007). EGF receptor ubiquitination is not necessary for its internalization.. Proc Natl Acad Sci U S A.

[31] Goh LK, Huang F, Kim W, Gygi S, Sorkin A Multiple mechanisms collectively regulate clathrin-mediated endocytosis of the epidermal growth factor receptor.. J Cell Biol.

[32] Katzmann DJ, Odorizzi G, Emr SD (2002). Receptor downregulation and multivesicular-body sorting.. Nat Rev Mol Cell Biol.

[33] Mosesson Y, Shtiegman K, Katz M, Zwang Y, Vereb G (2003). Endocytosis of receptor tyrosine kinases is driven by monoubiquitylation, not polyubiquitylation.. J Biol Chem.

[34] Huang F, Kirkpatrick D, Jiang X, Gygi S, Sorkin A (2006). Differential regulation of EGF receptor internalization and degradation by multiubiquitination within the kinase domain.. Mol Cell.

[35] Umebayashi K, Stenmark H, Yoshimori T (2008). Ubc4/5 and c-Cbl continue to ubiquitinate EGF receptor after internalization to facilitate polyubiquitination and degradation.. Mol Biol Cell.

[36] Wang Y, Roche O, Yan MS, Finak G, Evans AJ (2009). Regulation of endocytosis via the oxygen-sensing pathway.. Nat Med.

[37] Duan L, Miura Y, Dimri M, Majumder B, Dodge IL (2003). Cbl-mediated ubiquitinylation is required for lysosomal sorting of epidermal growth factor receptor but is dispensable for endocytosis.. J Biol Chem.

[38] Maxwell PH, Wiesener MS, Chang GW, Clifford SC, Vaux EC (1999). The tumour suppressor protein VHL targets hypoxia-inducible factors for oxygen-dependent proteolysis.. Nature.

[39] Sebastian S, Settleman J, Reshkin SJ, Azzariti A, Bellizzi A (2006). The complexity of targeting EGFR signalling in cancer: from expression to turnover.. Biochim Biophys Acta.

[40] Sorkin A, Goh LK (2008). Endocytosis and intracellular trafficking of ErbBs.. Exp Cell Res.

[41] Longva KE, Blystad FD, Stang E, Larsen AM, Johannessen LE (2002). Ubiquitination and proteasomal activity is required for transport of the EGF receptor to inner membranes of multivesicular bodies.. J Cell Biol.

[42] Geng Y, Kohli L, Klocke BJ, Roth KA Chloroquine-induced autophagic vacuole accumulation and cell death in glioma cells is p53 independent.. Neuro Oncol.

[43] Xu P, Duong DM, Seyfried NT, Cheng D, Xie Y (2009). Quantitative proteomics reveals the function of unconventional ubiquitin chains in proteasomal degradation.. Cell.

