# Supplementary figures and images for: The von Hippel-Lindau Tumor Suppressor Protein Promotes c-Cbl-Independent Poly-Ubiquitylation and Degradation of the Activated EGFR
Source: PLoS One. 2011 Sep 16;6(9):e23936. doi: 10.1371/journal.pone.0023936 (PMC3174936; doi:10.1371/journal.pone.0023936)

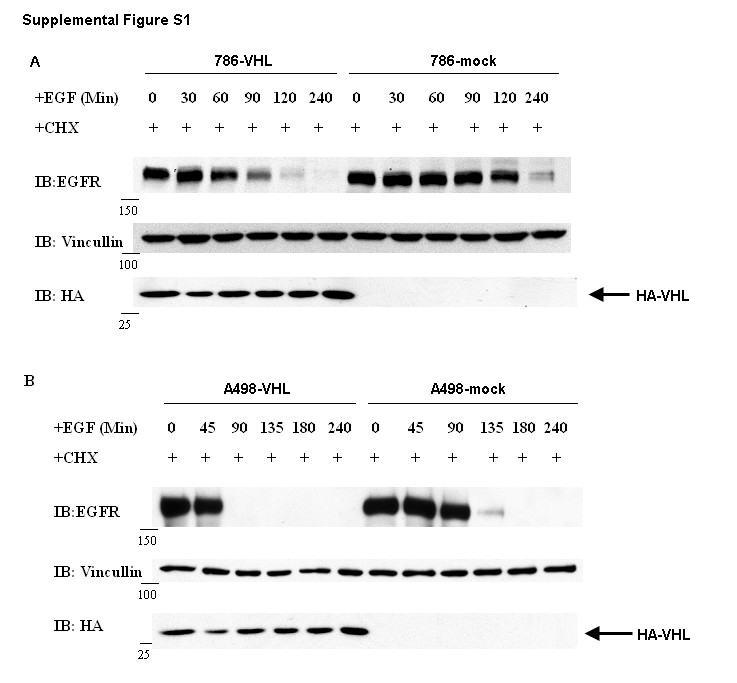

Supplement: Figure S1 — Activated EGFR had higher stability in VHL-deficient ccRCC cells than in VHL-expressing ccRCC cells in the presence of cycloheximide. A. Renal carcinoma 786-O cells transfected to produce wild type HA-VHL (786-VHL) or with an empty plasmid (786-mock) were starved for two hours in serum free DMEM media in the presence of 100 µg/ml cycloheximide (CHX) before addition of 30 ng/ml EGF. Total cell lysates were prepared at indicated time points and immunoblotted with the indicated antibodies. B. The same experiment in Fig. S1A was repeated with human renal carcinoma A498 cell lines with or without HA-VHL. (TIF) [file pone.0023936.s001.tif]

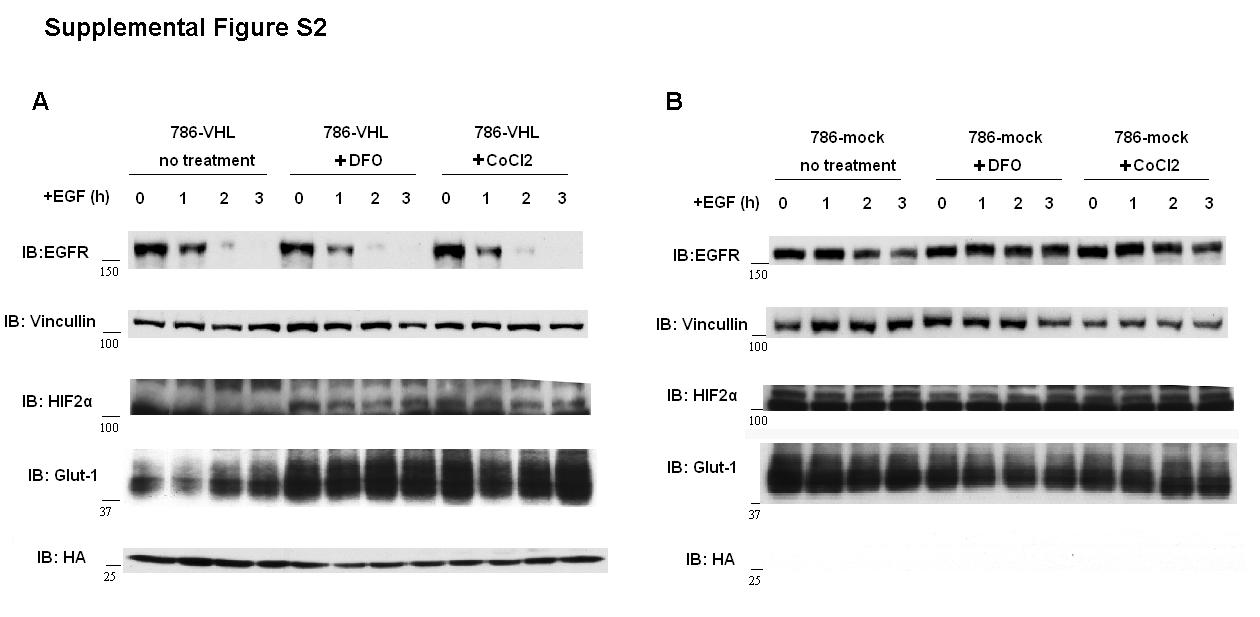

Supplement: Figure S2 — Hypoxia mimetics did not significantly increase the half-lives of activated EGFR in VHL-expressing ccRCC cells. 786-VHL (A) and 786-mock (B) cells were either untreated or treated with 100 µM hypoxia mimetics (DFO or CoCl2) for twenty-two hours. Then the cells were starved of serum for two hours in the absence or the presence of chemicals before the addition of 30 ng/ml EGF. The lysates were prepared with EBC buffer at indicated time and immunoblotted with indicated antibodies. Anti-HA blots detected HA-VHL. (TIF) [file pone.0023936.s002.tif]

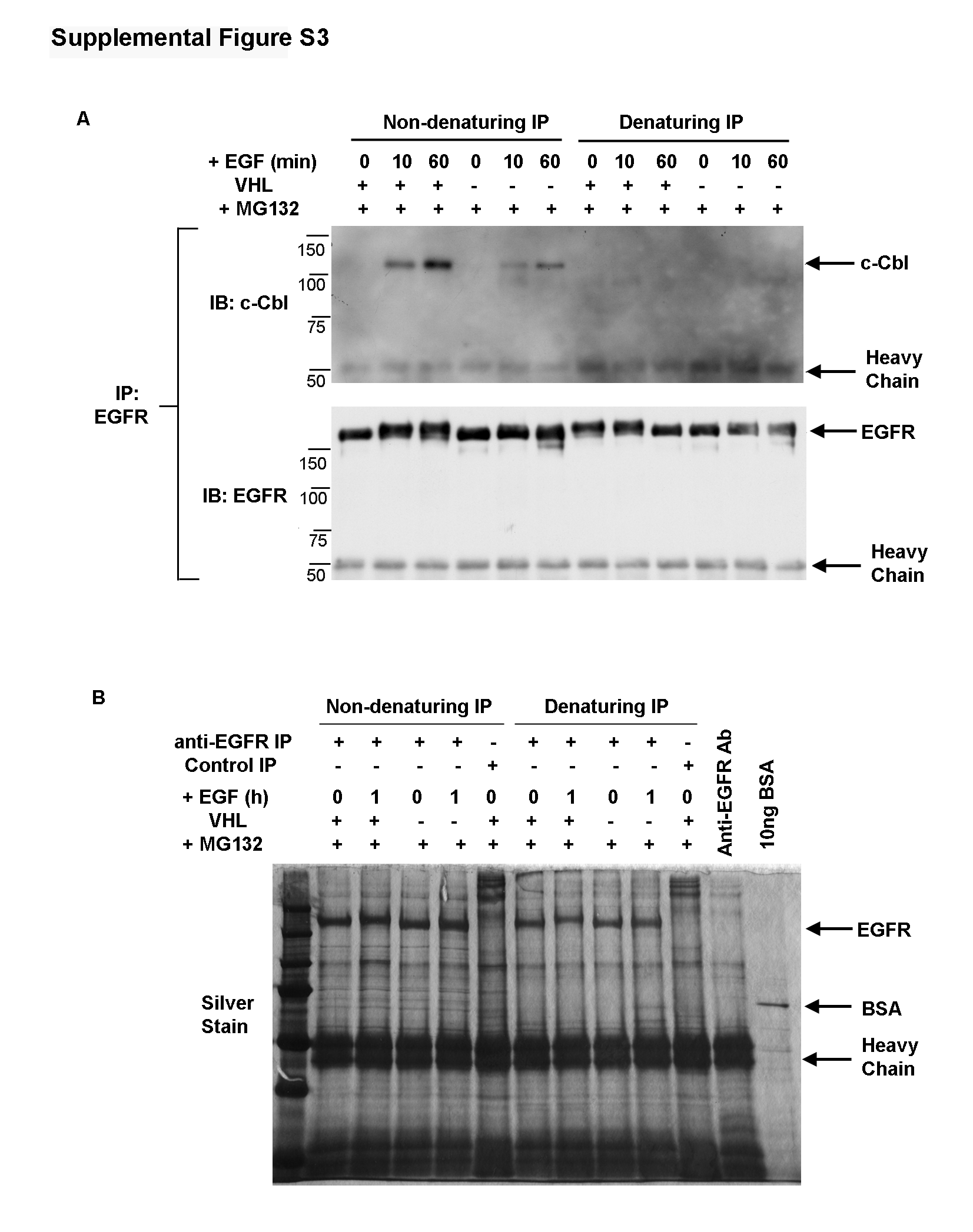

Supplement: Figure S3 — Denaturing IP removed proteins associated with activated EGFR. A. 786-VHL and 786-mock cells were starved and treated with 10 µM MG132 for two hours before addition of EGF. EBC lysates were generated. Half of the lystates were used for non-denaturing IP with anti-EGFR antibody, and the other half used for denaturing IP. The immunoprecipitated materials were blotted with indicated antibodies. B. A similar experiment as described in S3A was performed and the immunoprecipitated materials were resolved on a SDS-PAGE gel before silver staining. (TIF) [file pone.0023936.s003.tif]

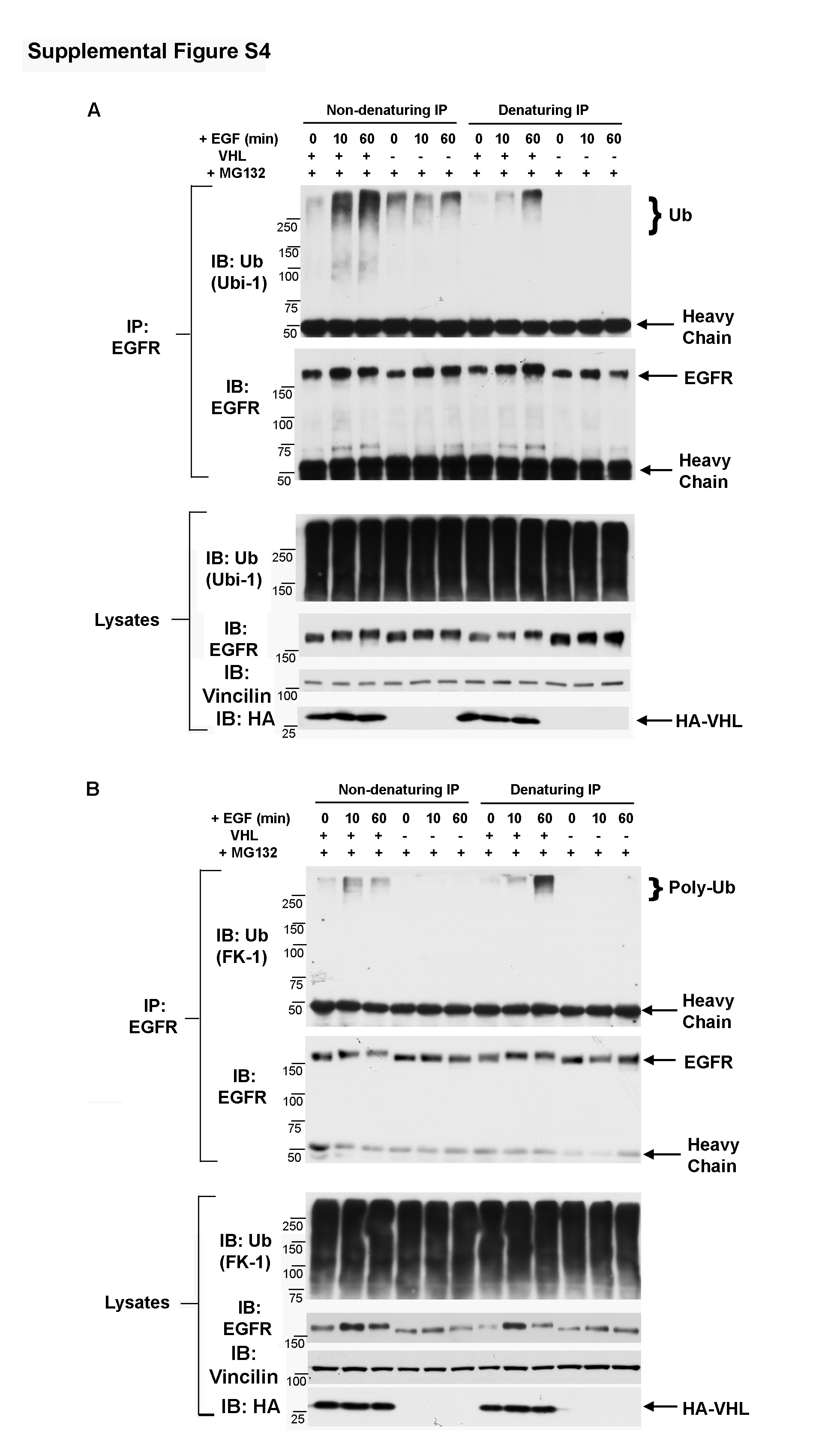

Supplement: Figure S4 — pVHL-dependent poly-ubiquitylation on the activated EGFR persisted after denaturing IP. A. 786-VHL and 786-mock cells expressing c-Cbl-1404 to avoid the interference of P4D1-specific Ub signals were starved of serum for two hours in the presence of 10 µM MG132 before the addition of 30 ng/ml EGF. The lysates were prepared with EBC buffer at indicated time. Half of the lysates were used for non-denaturing IP and the other half were used for denaturing IP. The immunoprecipitates were blotted with anti-Ub (Ubi-1) and anti-EGFR sequentially with membrane stripping between blots. The lysates were blotted with indicated antibodies. B. The same experiment as in S4A was performed and the immunoprecipitates were blotted with anti-poly Ub (FK-1) and anti-EGFR sequentially with membrane stripping between blots. The lysates were blotted with indicated antibodies. (TIF) [file pone.0023936.s004.tif]
